# Recent Developments in Rare Ovarian Carcinosarcoma: Literature Review and Case Report

**DOI:** 10.3390/diseases13060163

**Published:** 2025-05-22

**Authors:** Alexandra Nienhaus, Elena Bernad

**Affiliations:** 1 Doctoral School, “Victor Babes” University of Medicine and Pharmacy, Eftimie Murgu Square 2, 300041 Timisoara, Romania; 2 Department of Obstetrics and Gynaecology “Augusta Krankenanstalt” Bochum, Bergstr. 26, 44807 Bochum, Germany; 3 Department of Obstetrics and Gynecology, “Victor Babes” University of Medicine and Pharmacy, Eftimie Murgu Square 2, 300041 Timisoara, Romania; bernad.elena@umft.ro; 4 Center for Laparoscopy, Laparoscopic Surgery and In Vitro Fertilization, “Victor Babes” University of Medicine and Pharmacy, Eftimie Murgu Square 2, 300041 Timisoara, Romania

**Keywords:** ovarian carcinosarcoma, tubo-ovarian carcinosarcoma, case report, PARP inhibitors, HRD, advanced-stage ovarian cancer, carcinosarcoma complications

## Abstract

**Background and Objectives:** Ovarian carcinosarcoma (OCS) is a rare gynecologic malignancy defined by both epithelial and mesenchymal components, generally associated with advanced clinical stage and poor outcomes. We present a 66-year-old patient initially presenting with right iliac vein thrombosis, ultimately diagnosed with OCS, and place these findings in context with a focused literature review from 2000 through to 2024. **Methods:** A comprehensive account of the patient’s clinical course—spanning diagnostic imaging, surgical pathology, neoadjuvant chemotherapy, and interval debulking—was combined with a review of the current data on OCS pathogenesis, treatment protocols, and outcomes. **Results:** The patient’s tumor showed predominantly sarcomatous histology (approximately 90%) with high-grade serous features, responded to platinum/taxane chemotherapy, and was resected to no visible residual disease. The updated literature indicates that the majority of OCS cases present at advanced stages (often exceeding 60%), with suboptimal cytoreduction closely tied to worse prognosis. Up to 64% of tumors may harbor homologous recombination deficiency, offering a rationale for PARP inhibitor therapy; nonetheless, five-year survival rarely surpasses 45% in most series. **Conclusions:** Despite its aggressive course, optimal debulking surgery plus platinum-based chemotherapy remain central in treating OCS. Emerging molecular insights highlight homologous recombination deficiency and BRCA mutations as potential therapeutic targets. Multidisciplinary care and future prospective studies are key to improving long-term outcomes in this challenging malignancy.

## 1. Introduction

Ovarian carcinosarcoma (OCS), also historically referred to as malignant mixed Müllerian tumor, is a rare yet notably aggressive ovarian malignancy that accounts for only a small fraction of all ovarian neoplasms but exhibits a distinctively poor prognosis in clinical practice. Epidemiological estimates suggest that OCS comprises up to 1–4% of all ovarian tumors, although the exact incidence varies due to relatively small sample sizes and the tendency to combine carcinosarcomas with other histological types in larger epidemiologic reports [1,2]. Patients most commonly present in their sixth or seventh decade of life, often with advanced-stage disease at the time of initial diagnosis, paralleling findings in high-grade serous ovarian carcinoma but exhibiting even lower survival rates [3,4].

OCS is characterized by the coexistence of carcinomatous (epithelial) and sarcomatous (mesenchymal) components within the same tumor, reflecting its “mixed” origin. While the epithelial portion is frequently high-grade serous, alternative histologic patterns—such as endometrioid or clear cell—have been described in smaller series [5,6]. The sarcomatous segment may appear homologous (e.g., spindle cell resembling fibrosarcoma or leiomyosarcoma) or heterologous (e.g., chondrosarcoma, rhabdomyosarcoma), underscoring the significant morphologic diversity inherent to OCS [7]. Collectively, this dual tissue phenotype complicates diagnosis and potentially influences treatment outcomes.

Although limited data exist compared to more prevalent epithelial ovarian cancers, mounting evidence indicates that OCS may share certain molecular alterations with high-grade serous carcinoma. TP53 mutations, for instance, are often detected in OCS, suggesting a fundamental role in disease pathogenesis [8,9]. Additionally, emerging studies indicate that some OCS tumors harbor homologous recombination pathway alterations—such as BRCA1 or BRCA2 mutations—raising the possibility of using targeted therapies like poly (ADP-ribose) polymerase (PARP) inhibitors [10,11]. While further research is necessary to determine the precise clinical significance of these findings, such insights represent a step toward more personalized treatment strategies for patients with this rare malignancy.

Clinically, the presentation of OCS can closely mirror that of other advanced ovarian cancers. Patients commonly report vague abdominal or pelvic pain, fullness, bloating, or increasing girth due to ascites. These nonspecific symptoms often contribute to delayed diagnoses and the frequent discovery of extensive intra-abdominal or distant metastases at the time of initial workup [12,13]. Cross-sectional imaging—often via computed tomography—remains essential for delineating tumor spread and guiding surgical planning, although the definitive confirmation of OCS hinges on histopathological examination. Notably, some patients may present with atypical clinical scenarios, including paraneoplastic phenomena, complex fistulizing disease, or rapidly progressing metastases, all reflective of the tumor’s aggressive biology [14].

The management of OCS generally parallels that of high-grade epithelial ovarian cancer, prioritizing maximal surgical cytoreduction followed by platinum-based chemotherapy. An optimal surgical debulking—defined by the absence of macroscopic residual disease—has long been associated with improved survival, although the scarcity of prospective trials specific to OCS makes it difficult to identify definitive prognostic factors [15,16]. Agents such as paclitaxel and carboplatin are most commonly employed, and salvage regimens may include gemcitabine, ifosfamide, or other second-line treatments, although their efficacy in OCS is not as well substantiated as in pure epithelial counterparts [17,18]. Recently, evidence for the role of PARP inhibitors in homologous recombination-deficient or BRCA-mutated OCS has surfaced in case series, expanding the therapeutic armamentarium available to selected patients.

Outcomes in OCS remain guarded compared to more common epithelial ovarian cancers, primarily owing to frequent advanced-stage presentations and the tumor’s aggressive course [19]. Even with comprehensive surgical management and multi-agent chemotherapy, five-year survival rates for advanced OCS are generally lower than those of high-grade serous ovarian carcinoma at similar stages [2]. Nonetheless, as new discoveries emerge regarding the molecular underpinnings of these mixed tumors, individualized treatment plans combining standard surgery, platinum-based chemotherapy, and newer targeted therapies, such as PARP inhibitors, may improve the prognosis for a subset of patients [20].

Therefore, we detail an unusual case of a patient with ovarian carcinosarcoma. Additionally, we aimed to synthesize the major insights identified in recent publications on OCS, focusing on the evolving role of molecular testing (HRD, BRCA status), the potential benefit of PARP inhibitors in select patients, and the pressing need for prospective multicenter research for ovarian carcinosarcoma. It is hoped that this newly presented information will help guide clinicians in recognizing the diverse presentations of OCS, advocating for maximal cytoreduction when feasible, and utilizing emerging targeted therapies for this historically under-studied tumor type.

## 2. Materials and Methods

### 2.1. Case Identification and Ethical Considerations

Our index case was identified through the gynecologic oncology service of our institution after a 66-year-old female presented with signs of right lower extremity venous obstruction. She reported persistent swelling, pain, and edema in the proximal thigh region. Duplex ultrasound confirmed thrombosis of the right common and external iliac veins. CT imaging subsequently revealed a presacral mass, suggesting possible infiltration into nearby vascular structures.

Prior to publishing this case report, we obtained the patient’s written informed consent for using deidentified clinical data and images in accordance with the institutional guidelines and the Declaration of Helsinki. Ethical approval for retrospective analysis was also obtained from our local ethics committee. Safeguards were in place to ensure patient anonymity, and no personally identifying details are disclosed.

### 2.2. Literature Review

To contextualize the case, we conducted a focused review of English-language OCS publications from 1 January 2000 through to 31 December 2024 that specifically addressed ovarian carcinosarcoma. Data extracted from each publication included sample size, study design (case series, registry-based analysis, or single case), clinical presentation, molecular findings (e.g., HRD testing, BRCA status), therapeutic modalities (surgery, chemotherapy, targeted agents), and survival or patient outcomes, where available. We excluded any articles that predominantly focused on uterine carcinosarcoma or combined both uterine and ovarian carcinosarcoma data without specifying the subset for OCS. Case reports discussing purely in vitro findings without clinical correlation were also omitted.

### 2.3. Data Extraction and Synthesis

Data collection involved a comprehensive review of electronic health records and patient charts to extract relevant information. Variables collected included age at diagnosis, body mass index (BMI), histological type, grade of the tumor, stage at presentation, type of surgical treatment, adjuvant therapies (radiotherapy and chemotherapy), recurrence, and survival status at last follow-up.

Two investigators independently read each full-text article and abstracted the relevant clinical and pathological details. Given the heterogeneous nature of OCS research—ranging from single case reports to retrospective institutional case series—a consistent data extraction template was utilized to capture the following: (1) study type and sample size: single-patient case reports vs. multi-patient cohorts; (2) patient demographics: age, comorbidities, family history (if relevant); (3) clinicopathological features: disease stage, presenting symptoms, tumor size, histologic details (heterologous vs. homologous sarcomatous components); (4) molecular insights: HRD positivity, BRCA status (germline vs. somatic), mismatch repair, and other biomarkers; (5) therapeutic interventions: extent of surgery (optimal vs. suboptimal cytoreduction), chemotherapy regimens (platinum-based, taxanes, or others), and additional therapies such as PARP inhibitors; and (6) outcomes: progression-free survival (PFS), overall survival (OS), and presence of complications (e.g., paraneoplastic syndromes, fistulization, metastases).

Discrepancies in data interpretation were resolved by consensus. Only findings explicitly stated in these eight references were included, consistent with our objective of avoiding speculation or introducing external variables not reported by these studies. A narrative synthesis of the extracted information was performed.

### 2.4. Diagnostic Protocol and Pathological Review

The diagnosis of OCS requires histopathological confirmation of coexisting malignant epithelial (carcinomatous) and mesenchymal (sarcomatous) components. In our patient, initial suspicion arose from imaging findings of a large pelvic mass intimately associated with vascular structures, combined with significantly elevated tumor markers (CA 125, CA 15-3, CA 72-4). Surgical sampling (laparoscopic biopsy of presacral tissue and peritoneal washings) is essential to defining the lesion’s histology.

Clinically, the overlap of morphological variants within the same tumor underscores why pathologists must thoroughly sample suspected OCS. Heterologous elements (e.g., chondrosarcoma, rhabdomyosarcoma) may occur, further complicating the classification. A meticulous approach—encompassing histology, immunohistochemistry, and molecular profiling—thus forms the cornerstone of accurate diagnosis and informs targeted treatment decisions, especially as novel agents (e.g., PARP inhibitors, immunotherapy) are integrated into the treatment paradigm for OCS.

### 2.5. Treatment Algorithm and Follow-Up

The pillars of therapy include the following: (1) Surgical cytoreduction: achieving no visible residual disease (R0 resection) is a major prognostic factor. Where up-front optimal resection is not achievable due to extensive tumor burden, neoadjuvant chemotherapy followed by interval debulking is often pursued. (2) Chemotherapy: platinum-based regimens (carboplatin ± paclitaxel) are standard, with some institutions adding bevacizumab. Heterologous sarcomatous components may diminish chemosensitivity, but published case reports still document partial or complete responses in advanced stages. (3) Maintenance therapy: in patients harboring BRCA mutations or HRD positivity, PARP inhibitors (olaparib, niraparib) may extend progression-free intervals. This approach parallels the practice in high-grade serous carcinoma, although robust prospective evidence specific to OCS is limited. (4) Adjuvant and palliative therapies: some patients with advanced or metastatic disease may require additional measures, including palliative surgeries to relieve GI obstruction or the drainage of malignant effusions.

## 3. Results

### Case Report

**Figure 1:**
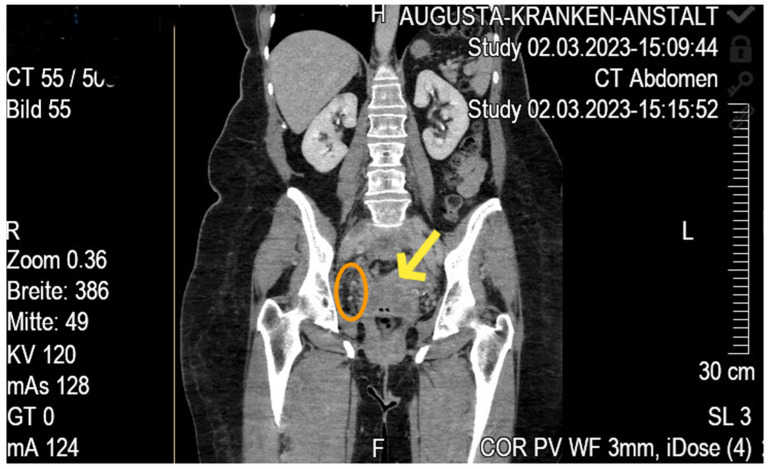
Contrast-enhanced axial CT of the abdomen and pelvis. Yellow arrow = 7 cm hyper dense presacral mass compressing right external iliac vein; dashed circle = cluster of collateral veins in the inguinal region indicating venous outflow obstruction.

A 66-year-old woman presented with right lower extremity edema and discomfort. Duplex ultrasound identified thrombosis within the right common and external iliac veins. A subsequent CT scan revealed a 7 cm presacral mass with prominent contact to bilateral iliac vessels, more extensive on the right side, consistent with an advanced pelvic neoplasm. Collateral vessels in the right inguinal region and ventral lower abdomen indicated significant venous outflow compromise, leading to edema and swelling of the proximal thigh. Imaging also showed possible fibroids, an inhomogeneous cervix, and a left ovarian cyst (Figure 1).

Laboratory workup, including serum tumor markers, suggested malignant behavior with elevations in CA 125, CA 15-3, and CA 72-4 (serum tumor markers were markedly elevated: CA 125 at 409 U/mL, CA 15-3 at 45.6 U/mL, and CA 72-4 at 48.8 U/mL).

**Figure 2:**
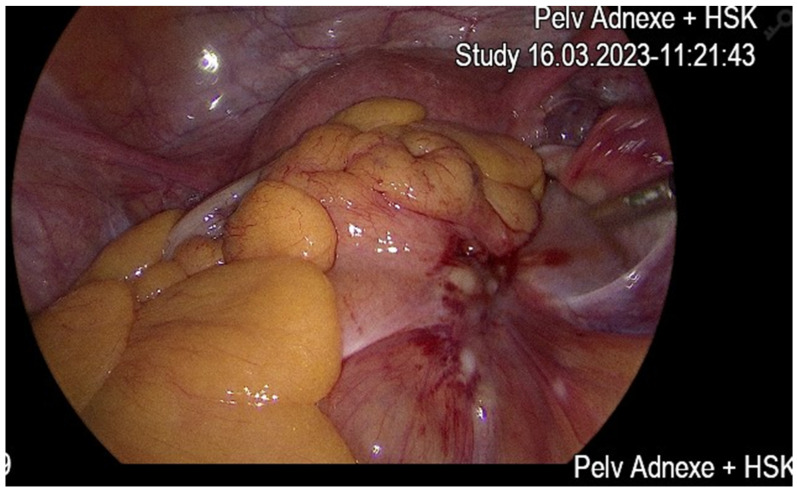
Laparoscopy intra-operative view.

Surgical hysteroscopy with polypectomy and conization was unrevealing. However, laparoscopic assessment (Figure 2), including peritoneal washings and right-sided presacral biopsy, identified an ovarian-based carcinoma with p16 positivity.

Upon histological examination, the epithelial portion showed high-grade serous morphology with p16 positivity, described as “The epithelial component shows high grade serous morphology with diffuse p16 expression, whereas the sarcomatous areas exhibit malignant spindle cells strongly positive for vimentin and WT 1 but negative for desmin”. Meanwhile, the sarcomatous areas displayed malignant spindle cells occupying approximately 90% of the tumor mass, an aggressive configuration paralleling heterologous features. Immunohistochemistry confirmed dual differentiation: pan-cytokeratin AE1/AE3 (+) and p53 mutant pattern, while MMR proteins were not tested.

**Figure 3:**
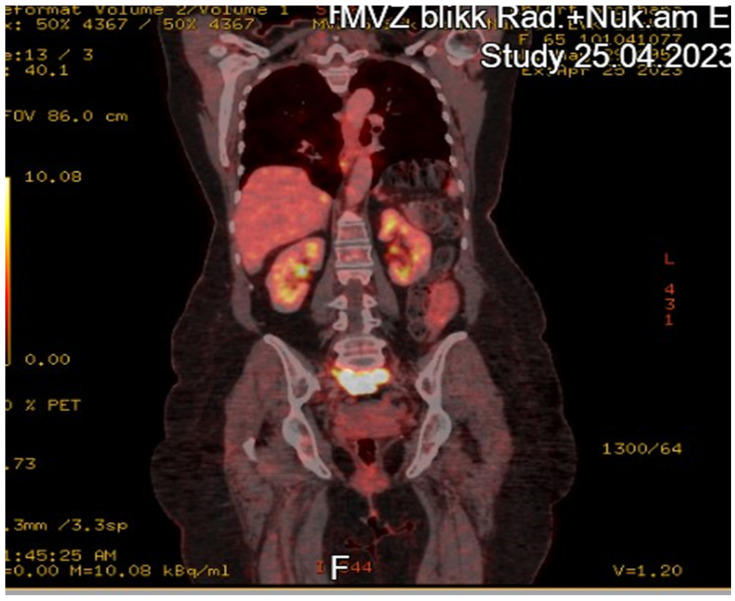
Coronal ^18F FDG PET CT demonstrating intense hyper metabolism (SUVmax = 13.2) of the presacral carcinosarcoma with encasement of the right common iliac vein.

With advanced disease, the multidisciplinary team recommended neoadjuvant carboplatin/paclitaxel for two cycles, aiming to reduce tumor burden before attempting radical resection. Follow-up imaging indicated partial response. The patient underwent an interval debulking surgery at a tertiary center, encompassing total hysterectomy, bilateral salpingo-oophorectomy, infragastric omentectomy, splenectomy, and the excision of the involved peritoneum. Notably, no residual macroscopic tumor was left (R0). Postoperatively, she completed six total cycles of chemotherapy. Pathology of the resected presacral mass again confirmed OCS with 90% sarcomatous predominance. Following treatment, a PET-CT scan was performed, as presented in Figure 3.

Table 1 provides a summary of recent publications on ovarian carcinosarcoma (OCS) [21,22,23,24,25,26,27,28]. The studies range from single case reports (St Laurent et al. [22], Wang et al. [23], Sumino et al. [24], Siala et al. [25], Elemian et al. [26], Yoriki et al. [27]) to larger cohort analyses (Fan Liang et al. [21] with 51 cases and Ha et al. [28] examining 458 patients from a national registry).

First, advanced stage at diagnosis is consistently highlighted. Wang et al. [23] and St Laurent et al. [22] both report stage IVB presentations with distant metastases—lung involvement in Wang’s case and inguinal node disease in St Laurent’s. Even in national registry data (Ha et al. [28]), a substantial proportion of OCS patients presented with regional or distant spread. Alongside advanced stage, suboptimal surgical debulking is repeatedly tied to poorer outcomes, reflecting the recognized significance of optimal cytoreduction in ovarian cancer.

Second, molecular profiling is progressively integrating into clinical care. Fan Liang et al. [21] identified a high prevalence of homologous recombination deficiency (64%) among OCS specimens. Although no patients exhibited microsatellite instability, HRD positivity opens up the door for PARP inhibitor maintenance, as exemplified by St Laurent et al. [22] (stage IVB BRCA1) and Yoriki et al. [27] (stage IIIB BRCA2). Both case reports documented encouraging responses or ongoing remissions. Notably, these accounts align with the broader shift in ovarian cancer management toward biomarker-driven therapies.

Third, the existing literature documents a diverse array of unusual clinical manifestations and complications. Siala et al. [25] described a sigmoido-ovarian fistula complicating OCS, necessitating emergent surgery. Elemian et al. [26] presented the first known instance of advanced OCS as non-islet cell tumor hypoglycemia, while Wang et al. [23] highlighted persistent fever related to lung metastases. These dramatic scenarios underscore the tumor’s propensity for aggressive, atypical behavior. Meanwhile, Sumino et al. [24] reported an early-stage (T1C) OCS arising from an endometriotic cyst, achieving prolonged (3-year) recurrence-free survival with docetaxel/carboplatin.

Finally, the nationwide data from Ha et al. [28] confirm OCS’s rarity (1.5% of epithelial ovarian cancers) and reveal a 5-year overall survival rate around 42.5%, echoing the grave prognosis. The synergy between these findings highlights the complexity of OCS: while multi-modality treatment (aggressive surgery + chemotherapy) remains foundational, emerging molecular insights (HRD, BRCA testing) and new therapies (PARP inhibitors) offer hope for improving outcomes in a historically intractable disease.

Table 2 specifically focuses on two multi-patient analyses from 2024, Fan Liang et al. [21] and Ha et al. [28], which together represent the largest cohorts of ovarian carcinosarcoma (OCS) reported in the recent literature. While Fan Liang et al. [21] assembled 51 cases from a single or limited set of institutions, Ha et al. [28] reviewed data from the Korean National Cancer Registry, encompassing 458 patients diagnosed over a nearly 20-year window. Both investigations highlight key clinicopathological characteristics that deepen our collective understanding of OCS.

A major unifying theme is the predominance of advanced-stage disease: 65% in Fan Liang et al. [21] and 65% distant disease in Ha et al. [28]. Notably, this advanced stage at presentation correlates with a grim prognosis, with Ha et al. [28] documenting a median overall survival (OS) of 39 months across their nationwide sample. The smaller series from Fan Liang et al. [21] showed a slightly higher median OS of 40 months, though it is important to note that these cohorts were not identical in demographic or treatment patterns.

A novel contribution from Fan Liang et al. [21] was the comprehensive molecular profiling, revealing that 64% of OCS tumors harbored homologous recombination deficiency (HRD) and 0% had microsatellite instability (MSI). This suggests a unique genetic landscape distinct from other gynecologic malignancies, potentially expanding the therapeutic windows for PARP inhibitor use. Ha et al. [28] did not specifically evaluate molecular markers, but they did find that younger patients (<50 years) had better survival than older individuals, echoing trends seen in other ovarian cancer subtypes.

Table 3 describes the emerging molecular insights and targeted therapeutic approaches. Fan Liang et al. [21] offer the most extensive molecular dataset: 64% of their 51 OCS cases tested positive for HRD, with none displaying microsatellite instability (MSI) and overall low tumor mutational burden (TMB). This genetic signature is unique and sets a foundation for exploring PARP inhibitors, widely used in high-grade serous ovarian cancer, harboring similar defects in DNA repair pathways. The single-patient reports by St Laurent et al. [22] and Yoriki et al. [27] underscore the potential real-world impact of this approach: both involved advanced OCS in patients with germline BRCA mutations (BRCA1 and BRCA2, respectively). After debulking surgery and platinum-based chemotherapy, each received PARP inhibitor maintenance, achieving durable remission, one at 25 months and the other still ongoing.

Conversely, in the case of Elemian et al. [26] (where no molecular testing was mentioned), the patient’s OCS rapidly progressed despite platinum-based chemotherapy, culminating in death. While multiple confounders could explain this unfortunate outcome, the absence of targeted therapies or knowledge of HRD/BRCA status stands out. This dichotomy underscores how early identification of targetable mutations (e.g., BRCA) might shift prognosis, particularly for advanced-stage OCS.

Sumino et al. [24], reporting on an early-stage (T1C) heterologous OCS, did not provide molecular data but highlighted how standard chemotherapy with docetaxel/carboplatin achieved an impressive 3-year recurrence-free interval. This scenario suggests that robust outcomes are possible even without targeted agents when the disease is detected early and is fully resected. Finally, the national registry study by Ha et al. [28] reaffirms that OCS rarely surpasses 40–42% five-year overall survival. The integration of molecular diagnostics into large registries could offer invaluable insights into the prevalence of HRD or BRCA changes and might eventually shift these survival statistics in more favorable directions. Overall, the data strongly suggest that molecular profiling—especially for HRD/BRCA—could become a critical element in optimizing therapy for OCS (Table 4).

## 4. Discussion

### 4.1. Analysis of Findings

St Laurent et al. [22] presented a 36-year-old patient with stage IVB OCS and a BRCA1 mutation. Remarkably, she had minimal intraperitoneal spread but an inguinal lymph node metastasis and a serous tubal intraepithelial carcinoma. After standard debulking surgery and platinum-based chemotherapy, maintenance with a PARP inhibitor yielded extended remission beyond three years. This highlights how targeted therapy can potentially alter the grim outlook traditionally associated with OCS, especially in younger patients with germline BRCA mutations.

In the case by Wang et al. [23], a 61-year-old with stage IVB disease experienced persistent fever and metastatic pulmonary lesions. OCS was confirmed post-surgery, but disease rapidly progressed, culminating in death. This unfortunate scenario illustrates the aggressive course that advanced OCS can take, even when recognized and treated promptly. Moreover, in an early-stage T1C3 OCS arising from an endometriotic cyst, docetaxel plus carboplatin and prompt surgical management conferred a 3-year disease-free interval, as presented by Sumino et al. [24].

Siala et al. [25] presented a case of a 67-year-old with OCS complicated by a sigmoido-ovarian fistula, which exemplifies how the tumor’s rapid infiltration can breach organ planes, leading to emergent conditions like fistulization and peritonitis. Though radical resection was performed, disease progression was evident on postoperative imaging, underscoring the complexities in advanced OCS surgical management. Furthermore, Elemian et al. [26] described a 55-year-old patient who presented with non-islet cell tumor hypoglycemia, a rare paraneoplastic phenomenon, which severely complicated management. Despite high-dose glucose infusions and chemotherapy, she succumbed to multiorgan failure.

Finally, Yoriki et al. [27] presented a 43-year-old patient with stage IIIB disease carrying a germline BRCA2 mutation who also had concurrent ductal carcinoma in situ (breast). Postoperative chemotherapy followed by PARP inhibitor maintenance resulted in ongoing remission at 25 months. This case echoes the findings of Ha et al. [28] in demonstrating that targeted maintenance can be particularly impactful in BRCA-mutated OCS. Altogether, these reports illustrate the vast clinical variability of OCS, from early, potentially curable stages to advanced, rapidly fatal scenarios. They further emphasize that while standard debulking and platinum-based chemotherapy remain central, personalized strategies—especially incorporating PARP inhibitors for BRCA/HRD cases—are increasingly shaping OCS management. Other studies showed that PARP inhibitor maintenance yielded a median progression-free survival of 23.5 months vs. 17.3 months with placebo [29]. Additionally, clinical trials evaluated anti-PD-1 plus lenvatinib in WT-1-positive carcinosarcomas, although without significant findings [30].

In a similar manner, the study by Xu et al. [31] highlighted the clinical and imaging features of five cases of primitive OCS, which are rare tumors marked by both carcinomatous and sarcomatous components. Their findings noted that all patients presented with unilateral tumors, varying in size from 11 cm to 14 cm, and demonstrated diverse CT and MRI morphologies, such as solid masses with cystic areas or necrosis and multilocular cystic masses with solid protrusions. Despite the heterogeneity in presentation, four patients showed elevated CA 125 levels, and three had elevated CA 153 levels. Following surgical resection and adjuvant chemotherapy, three patients exhibited no obvious recurrence or metastasis during follow-ups ranging from 9 to 59 months, while one patient succumbed to the disease due to recurrence and metastasis. Contrastingly, McFarlane et al. [32] conducted a comprehensive multi-cohort cross-sectional study comparing ovarian carcinosarcoma with other histotypes across large Scottish and SEER cohorts. This study revealed that OCS patients were significantly older at diagnosis and consistently demonstrated the poorest survival rates, even in early stages of the disease compared to other histotypes. Specifically, within the Scottish cohort, a large percentage of OCS patients presented with advanced FIGO stage III and IV disease, and multivariable analysis indicated significantly shorter survival times for OCS compared to high-grade serous, endometrioid, and other forms, underscoring the aggressive nature of OCS and the urgent need for effective therapeutic strategies.

Beyond ubiquitous TP53/BRCA alterations, two emerging OCS selective signals deserve mention: over expression of N myristoyltransferase 1 (NMT1) identified in many ovarian tumors but <5% of high-grade serous controls [33] and focal CCND1 amplification associated with rapid proliferation and chemoresistance [34]. Their diagnostic and therapeutic relevance warrants prospective validation.

The case report on ovarian carcinosarcoma illustrates the clinical challenges and treatment nuances associated with this rare and aggressive cancer, often presenting at an advanced stage with poor outcomes. By detailing the diagnostic process, treatment regimen, and integration of neoadjuvant chemotherapy and optimal debulking surgery, this report highlights the clinical significance of early and aggressive treatment strategies to manage OCS. Furthermore, it brings attention to emerging research on molecular targets such as homologous recombination deficiency and BRCA mutations, underscoring their potential to enhance therapeutic precision and improve prognostic outcomes. This case not only enriches the existing literature on OCS but also underscores the importance of multidisciplinary care and the need for ongoing research to refine treatment approaches for this challenging malignancy.

### 4.2. Study Limitations and Future Perspectives

This manuscript is constrained by its reliance on recent publications and a single institutional case. Although these references yield valuable perspectives on the current state of OCS research, they do not capture the entire global experience or the broader historical context. However, older data might not be reliable, since oncology is a field that has changed dramatically over time. The inherent rarity and heterogeneity of OCS further complicate efforts to standardize management algorithms. Finally, the potential benefits of PARP inhibitors in OCS are promising but remain preliminary, highlighting a need for dedicated prospective trials.

## 5. Conclusions

Ovarian carcinosarcoma is a formidable malignancy characterized by advanced presentation, complex paraneoplastic phenomena, and a historically poor prognosis. Early histopathological diagnosis, aggressive surgical cytoreduction, and platinum-based chemotherapy constitute the foundation of contemporary treatment. Emerging molecular insights—especially related to HRD and BRCA mutations—suggest a widening role for PARP inhibitors in maintenance or relapse settings. The case presented here underscores both the heterogeneity of OCS and the incremental progress in identifying effective, personalized therapeutic strategies. Larger prospective trials and registry-based collaborations remain essential to improve long-term outcomes for patients with this rare and deadly disease.

## Figures and Tables

**Table 1:** Overview of ovarian carcinosarcoma studies.

Reference	Study Design/Sample	Key Focus	Main Findings
Fan Liang et al. [21]	Retrospective analysis of 51 tubo-ovarian carcinosarcomas	Clinicopathological and molecular profiles	65% advanced stage; suboptimal debulking → poor outcomes; 64% HRD+
St Laurent et al. [22]	Single case report, stage IVB OCS + BRCA1 mutation	Successful PARP inhibitor therapy	Complete response on olaparib; underscores role of BRCA-driven OCS
Wang et al. [23]	Single case report, stage IVB OCS with lung metastases	Persistent fever as initial presentation	Diagnosed postoperatively; patient succumbed to disease progression
Sumino et al. [24]	Single case report, T1C OCS in an endometriotic cyst	Rare, early-stage OCS, heterologous type	3-year recurrence-free survival after docetaxel/carboplatin
Siala et al. [25]	Single case report with complicated OCS (sigmoido-ovarian fistula)	Surgical management of GI involvement	Radical resection with Hartmann procedure; advanced disease on follow-up
Elemian et al. [26]	Single case report, advanced OCS with paraneoplastic hypoglycemia (NICTH)	Unusual endocrine manifestation	Despite chemo, patient died of multiorgan failure shortly after Dx
Yoriki et al. [27]	Single case report, germline BRCA2-associated OCS	Feasibility of maintenance PARP inhibitor	Stage IIIB OCS; ongoing remission at 25 months on PARP inhibitor
Ha et al. [28]	National registry (Korea): 458 OCS cases (1999–2018)	Epidemiology, incidence, and survival outcomes	1.5% of epithelial ovarian cancers; median OS 39 mo; 5-year OS ~42.5%

**Table 2:** Clinicopathological features of OCS.

Study	No. of Cases	Stage Distribution	Median PFS (Months)	Median OS (Months)	Key Clinicopathological Findings
Fan Liang et al. [21]	51	65% advanced (≥III)	27	40	− 64% HRD+ − Suboptimal debulking → poorer outcome − 0% MSI, low TMB
Ha et al. [28]	458	Localized 14%, Regional 21%, Distant 65%	Not specified	39	− Incidence rising over 20 yrs − 5-yr OS ~42.5% − Younger (<50) fare better

**Table 3:** Types of systemic therapy insights on OCS.

Reference	HRD/BRCA Status	Therapeutic Intervention	Outcome
Fan Liang et al. [21]	64% HRD+; 0% MSI, low TMB	Standard chemo (platinum-based)	HRD positivity suggests possible PARPi benefit
St Laurent et al. [22]	Germline BRCA1 mutation	Olaparib maintenance	Stage IVB → Clinical remission
Sumino et al. [24]	Not reported	Docetaxel/carboplatin	3-year recurrence-free survival (early-stage)
Elemian et al. [26]	Not specified; no testing described	Carboplatin/paclitaxel	Rapid deterioration; patient death
Yoriki et al. [27]	Germline BRCA2 mutation; HRD+ likely	PARPi (maintenance)	Ongoing remission at 25 months
Ha et al. [28]	No mention of HRD or BRCA specifically	Not detailed at molecular level	5-year OS ~42.5%; national dataset

**Table 4:** Summary of reported cases and outcomes.

Study	Patient Age	Stage	Unique Presentation/Complication	Management	Outcome
St Laurent et al. [22]	36	IVB	BRCA1 mutation, small STIC lesion, LN spread	CRS + Carboplatin/Taxane + PARPi	No recurrence at 3+ years
Wang et al. [23]	61	IVB	Persistent fever, lung metastases	Cytoreductive surgery + chemo	Rapid progression, patient died
Sumino et al. [24]	41	T1C3	OCS arising in endometriotic cyst	Secondary surgery + docetaxel/CBDCA	Recurrence-free at 40 months
Siala et al. [25]	67	T2b (est.)	Sigmoido-ovarian fistula + pelvic peritonitis	Emergent en-bloc resection (Hartmann)	Disease progression, scheduled for chemo
Elemian et al. [26]	55	Metastatic	NICTH (paraneoplastic hypoglycemia)	Glucose infusion + chemo	Deceased (multiorgan failure)
Yoriki et al. [27]	43	IIIB	Germline BRCA2, concurrent breast DCIS	CRS + platinum + PARPi maintenance	Alive, no recurrence at 25 months

CRS: cytoreductive surgery; CBDCA: carboplatin; LN: lymph node; DCIS: ductal carcinoma in situ; PARPi: PARP inhibitor.

## Data Availability

The data presented in this study are available on request from the corresponding author.
